# Feasibility of continuous fever monitoring using wearable devices

**DOI:** 10.1038/s41598-020-78355-6

**Published:** 2020-12-14

**Authors:** Benjamin L. Smarr, Kirstin Aschbacher, Sarah M. Fisher, Anoushka Chowdhary, Stephan Dilchert, Karena Puldon, Adam Rao, Frederick M. Hecht, Ashley E. Mason

**Affiliations:** 1 Department of Bioengineering and Halicioglu Data Science Institute, University of California, San Diego, 9500 Gilman Drive MC 0412, La Jolla, CA 92093-0412 USA; 2 Division of Cardiology, School of Medicine, University of California, San Francisco, San Francisco, USA; 3 Health Data Architect, Science Team, Oura, San Francisco, USA; 4 Osher Center for Integrative Medicine, University of California San Francisco, San Francisco, USA; 5 Department of Management, Baruch College, CUNY, New York and preValio LLC, Minneapolis, USA; 6 School of Medicine, University of California, San Francisco, San Francisco, USA; 7 Department of Psychiatry, University of California, San Francisco, San Francisco, USA

**Keywords:** Diagnostic markers, Predictive markers, Scientific data, Biotechnology

## Abstract

Elevated core temperature constitutes an important biomarker for COVID-19 infection; however, no standards currently exist to monitor fever using wearable peripheral temperature sensors. Evidence that sensors could be used to develop fever monitoring capabilities would enable large-scale health-monitoring research and provide high-temporal resolution data on fever responses across heterogeneous populations. We launched the TemPredict study in March of 2020 to capture continuous physiological data, including peripheral temperature, from a commercially available wearable device during the novel coronavirus pandemic. We coupled these data with symptom reports and COVID-19 diagnosis data. Here we report findings from the first 50 subjects who reported COVID-19 infections. These cases provide the first evidence that illness-associated elevations in peripheral temperature are observable using wearable devices and correlate with self-reported fever. Our analyses support the hypothesis that wearable sensors can detect illnesses in the absence of symptom recognition. Finally, these data support the hypothesis that prediction of illness onset is possible using continuously generated physiological data collected by wearable sensors. Our findings should encourage further research into the role of wearable sensors in public health efforts aimed at illness detection, and underscore the importance of integrating temperature sensors into commercially available wearables.

## Introduction

Fever is the first symptom listed in the Center for Disease Control and Prevention’s (CDC’s) “Quarantine and Isolation” informational site as of this writing^1^. There, fever is defined as “a measured temperature of 100.4 °F (38 °C) or greater, *or feels warm to the touch*” (our emphasis). Herein we assess data compared to subjective illness reports, and so use a more colloquial definition: Fever refers to an atypical elevation in body temperature generally (but not always) associated with an immunologic response to viral or bacterial infection^2^. Individual and government efforts to track the spread of SARS-CoV2 (in which infection is characterized by fever^3^) have employed classical thermometry at largely random timepoints, for example when entering a workplace or boarding a bus^4,5^. However, single-point measures have limited sensitivity to detect disease, particularly in the earliest stages of onset^6^; this lack of utility has dampened the perceived value of temperature assessment in disease prevention and containment. Further, some researchers have used the difficulties with single-point measures to argue that data from wearable devices cannot be used to detect fever^7^. This is not surprising, as sensitivity to detect small, but meaningful, changes in body temperature may be limited without contextual information, such as baseline variability in circadian body temperature^8^, phase in menstrual cycle^8–10^, and other temperature-modulating biological rhythms^8^ at the time of measurement. Without these contexts for measured individuals, inferring fever from a single-thermometry assessment is reliable only when the fever is well outside the range of these normal variations. It is likely that our current reliance on single-point temperature assessment has led to missed case identification, as indeed, the COVID-19 pandemic has not abated despite growing use of temperature checks, for example, upon entry to restaurants, stores, and air travel. Fundamentally, the question is about feasibility: Can continuous temperature observations allow us to extract information from temperature as a signal, and thereby overcome the barriers that have stymied efforts using single-point measurements?

Wearable sensor devices (wearables) equipped with temperature sensors could provide useful contextual information while assessing temperature, which we hypothesize would make temperature data more useful in fever detection. To our knowledge, no published data have shown that wearables might be a useful tool in fever identification (but see^11^ for comparison). Wearables that are consumer goods make a potential ready-to-use distributed monitoring system for changes to health within individuals and across whole populations. Evidence that wearables can detect fever should therefore be of interest to public health efforts, particularly in the context of pandemics.

We launched TemPredict in March of 2020 to assess whether we could identify the onset of COVID-19 symptoms using continuously collected, wearables-derived dermal temperature data from the Oura ring sensor device. No prior studies have demonstrated the feasibility of using such data to identify fevers. Although TemPredict is still ongoing, here we present early results from the first 50 subjects with enough data to meet analysis inclusion criteria. In these analyses, we demonstrate that fever detection and prediction via wearables is a promising avenue for research focused on improving fever tracking in the COVID-19 pandemic and future pandemics.

## Results

### Participants

Demographic and background information was available for all 50 participants. Most participants resided in the US (33; 66%), with an additional 6 participants in the UK, 3 in Finland, and 1 each in Austria, Canada, Germany, Honduras, Italy, The Netherlands, Norway, and Sweden. Of the 33 participants residing in the US, 11 lived in the state of California, 4 in New York, 3 in Florida, 2 each in New Jersey, North Carolina, and Washington, and 1 each in Massachusetts, Georgia, Minnesota, Illinois, Texas, Utah, and Oregon. Six participants (12%) indicated they worked at least 50% of their time in places where they are potentially in contact with patients seeking treatment for symptoms that characterize COVID-19. Of those 6 participants, 2 were attending physicians, 1 a nurse/nurse assistant, 2 were respiratory therapists, and 1 occupied another medical role. Most participants (66%) indicated their biological sex as male. Average age was 43.7 years (SD = 11.0, range 24–76, median = 40.5). Average household size was 3 (SD = 1.3, range 1–6, median = 3). Most participants held a 4-year college degree or higher (36% Bachelor’s, 26% Master’s, 12% professional [MD/JD etc.], 12% doctorate). Of the 48 participants who indicated their race or ethnicity, the majority (39; 81%) identified as Caucasian/White, 8 as Hispanic/Latino, 1 as Middle Eastern, 1 as Asian, 1 as South Asian, and 3 as “other” (then indicating they were of “European”, “Scandinavian”, or “Jewish” ethnicity). Of these 50, 35 had complete 65 day data windows (see “Methods”); 3 had ≤ 3 weeks prior; 2 had < 4 weeks prior; 2 had < 5 weeks prior; 1 had 6 weeks prior; 1 had 43 days prior.

### Is a single temperature value appropriate to identify fever using finger skin temperature?

**Figure 1:**
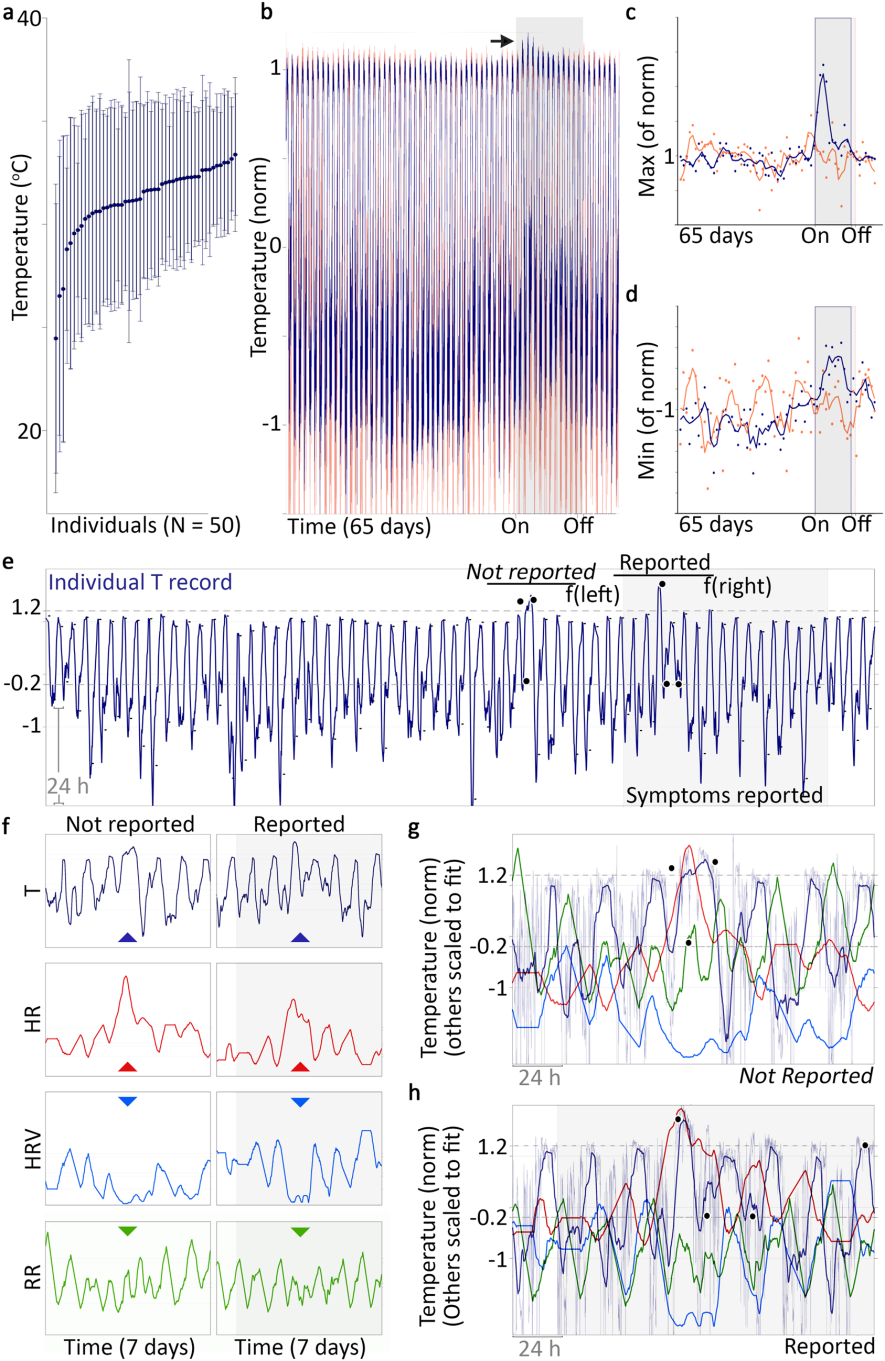
Wearable distal temperature sensors are suitable for developing digital biomarkers for fever with and without paired symptom reports. 50 individuals (**a**) display a wide range of temperatures (inter-individual means ± standard deviation) both during baseline (dark blue) and symptom report window (pail lines); there is a small but significant difference between the two sets of means. Having been normalized (norm) by individual range (see “Methods”), the mean 65-day temperature profile for individuals reporting fever (purple) and those not reporting fever (orange) reveal an apparent rise in maximum finger temperature (T) in fever-reporting cases near the beginning of the symptom window (black arrow; symptom window: grey box, mean duration for the whole population). Norm daily maximum (**c**) and minimum (**d**) T highlights changes in both that correspond to fever onset report. Such cases informed the construction of digital biomarkers in the form of thresholds for daily max and min that identify fever-like days. An example T record (**e**) with fever-like days identified by exceeding these thresholds before onset of symptom report (**e**; black dots represent daily min and max above thresholds) has similar changes in heart rate (HR), HR variability (HRV), and respiration rate (RR), to the reported fever event (**f** by variable, and **g**,**h** with overlay, respectively). (**f**–**h**) All lines are smoothed by 360 min radius, displaying the same smoothing used to generate median minimum and maximum values for each day. Faded blue line in (**g**,**h**) is the raw T (1 point/min).

We found substantial inter-individual variance in both mean and range (Fig. 1a; population mean ± standard deviation: 31.2 ± 1.7 °C). We observed a significant increase in T during the symptom window compared to baseline (Fig. 1a, dark and light lines, respectively; Wilcoxon rank-sum test of means by baseline vs symptom window, mean difference of + 0.63 ± 1 °C; *p* = 0.024). These findings support our hypothesis that wearables could be used to identify fever, while underscoring that the intra-individual variability is substantial enough that using a single temperature value (i.e. 38 °C) across all individuals would not be appropriate.

### Do wearable temperature time series show changes associated with fever self-reports?

Given our positive finding of mean increase T following symptom onset, we next assessed the data for signs that their continuous nature adds information related to reported fevers not easily assessed by single time-point measurements. Our first observation was that most intra-individual temperature variance is attributable to daily rhythms, with twice-daily large transitions between lower temperatures during the daytime (daily minimums), and higher temperatures across the night (daily maximums). Second, we noticed that participants who reported a fever (n = 38; 76%) also appeared to exhibit elevated maximum temperature concordant with symptom onset (Fig. 1b, dark line: mean temperatures across all 65 days for subjects reporting fever; orange line: mean temperatures across all 65 days for individuals not reporting a fever; grey box: average duration symptom window). Individuals not reporting fever (n = 12, 24%) did not show this rise in the sample average. Given these two observations, we generated representative “daily minimum” and “daily maximum” values across all days for all subjects, and normalized these further to allow uniform comparison (see “Methods”). Comparison within these minimum and maximum values provided clearer signals of temperature change associated with fever self-reports (Fig. 1c,d). The average daily maximum rose sharply and significantly as symptom onset approached (baseline vs 1st week of symptom window, rank sum test, with no fever-like days detected: *p* = 0.4; with fever-like days detected, *p* = 8 × 10^–6^), and the daily minimum showed a longer, more steady significant increase to a peak near reported symptom onset (baseline vs 1st week of symptom window, rank sum test, with no fever-like days detected: *p* = 0.4; with fever-like days detected, *p* = 2 × 10^–5^).

### Can observed patterns be used to develop digital biomarkers of probable fever?

Given the different behavior of nighttime and daytime temperature, we chose to create one biomarker for each extreme (see “Methods”). Because the point of this exploration was to test the feasibility of using wearable data for fever prediction and detection, and not to provide a global solution to fever monitoring (which would be inappropriate with such a small cohort), we did not develop biomarkers by machine learning (ML) or similar extraction. Rather, we assigned a threshold applied to the normalized daily minimum and maximum values to catch excursions associated with fever reports. We scored days exceeding either threshold (day or nighttime) as “fever-like” days. With these thresholds, 3/38 of those who reported fevers did not have fever-like days within their reported symptom window, whereas 7/12 who did not report fever nevertheless had fever-like days within their reported symptom window. To investigate when disagreements between self-report and digital biomarker-based detection might be due to imprecision of the digital biomarkers, and when they might instead be due to failure to notice or report fevers, we next compared to changes from other physiological variables at these detected episodes to assess the presence of coordinated disruptions that might reflect illness or related physiological disruption.

### Can appropriateness of digital biomarkers be assessed to guide future refinement?

When we re-sorted individuals into groups of those with (n = 42) and those without (n = 8) detected temperature-derived fever-like days during the symptom window, other physiological changes became more pronounced. Specifically, we observed larger and significant changes between the baseline and symptom windows for those with detected fever-like days, as compared to those with reported fever. Sorting individuals into groups using symptom report led to a non-significant difference between the baseline and the first week of reported symptoms (chosen because that is where the major temperature change was observed, on average; Supp 1) for HR and HRV (mean difference ± S.E.: HR: 0.49 ± 0.25 to 1.48 ± 0.28; HRV: − 0.15 ± 0.25 to − 0.42 ± 0.15; Tukey–Kramer post-hoc analysis following Kruskal–Wallis non-parametric comparison, bonferroni corrected; *p* = 0.13, *p* = 0.33, respectively). Unlike in HR and HRV, we did observe a significant difference in RR from baseline to during symptoms (− 0.12 ± 0.1 to 0.3 ± 0.06; *p* = 0.002), consistent with CDC reports that fever and respiratory distress are the two most common COVID-19 symptoms. By contrast, re-sorting individuals by digital biomarker resulted in significant differences between baseline and baseline and reported symptom windows in all three variables: HR, HRV, and RR (HR: 0.13 ± 0.28 to 1.45 ± 0.25, *p* = 0.02; HRV: 0.26 ± 0.13 to − 0.48 ± 0.14, *p* = 0.03; RR: − 0.06 ± 0.08 to 0.24 ± 0.07, *p* = 0.01). To summarize, baseline and symptom-onset HR and HRV were not significantly different in the population sorted by fever self-report, but were significantly different once sorted by temperature-derived digital biomarker.

### Can digital biomarkers be used to study the potential of illness detection before symptom report?

We applied our digital biomarkers across the 45 days prior to symptom onset. 38/50 subjects had fever-like days before reporting symptoms (e.g. Fig. 1e). To assess whether such events had other evidence of illness, as opposed to false positives, we examined other physiological outputs from the same time windows (heart rate, heart rate variability, and respiration rate). We visually inspected the changes across fever-like days before and during the reported symptom window, and found that many showed similar patterns to the change associated with fever detection just described (Fig. 1f,g): coordinated change across multiple physiological variables (T, HR, HRV, RR).

### If feasibility fever detection is so solid, where’s my flying car?

**Figure 2:**
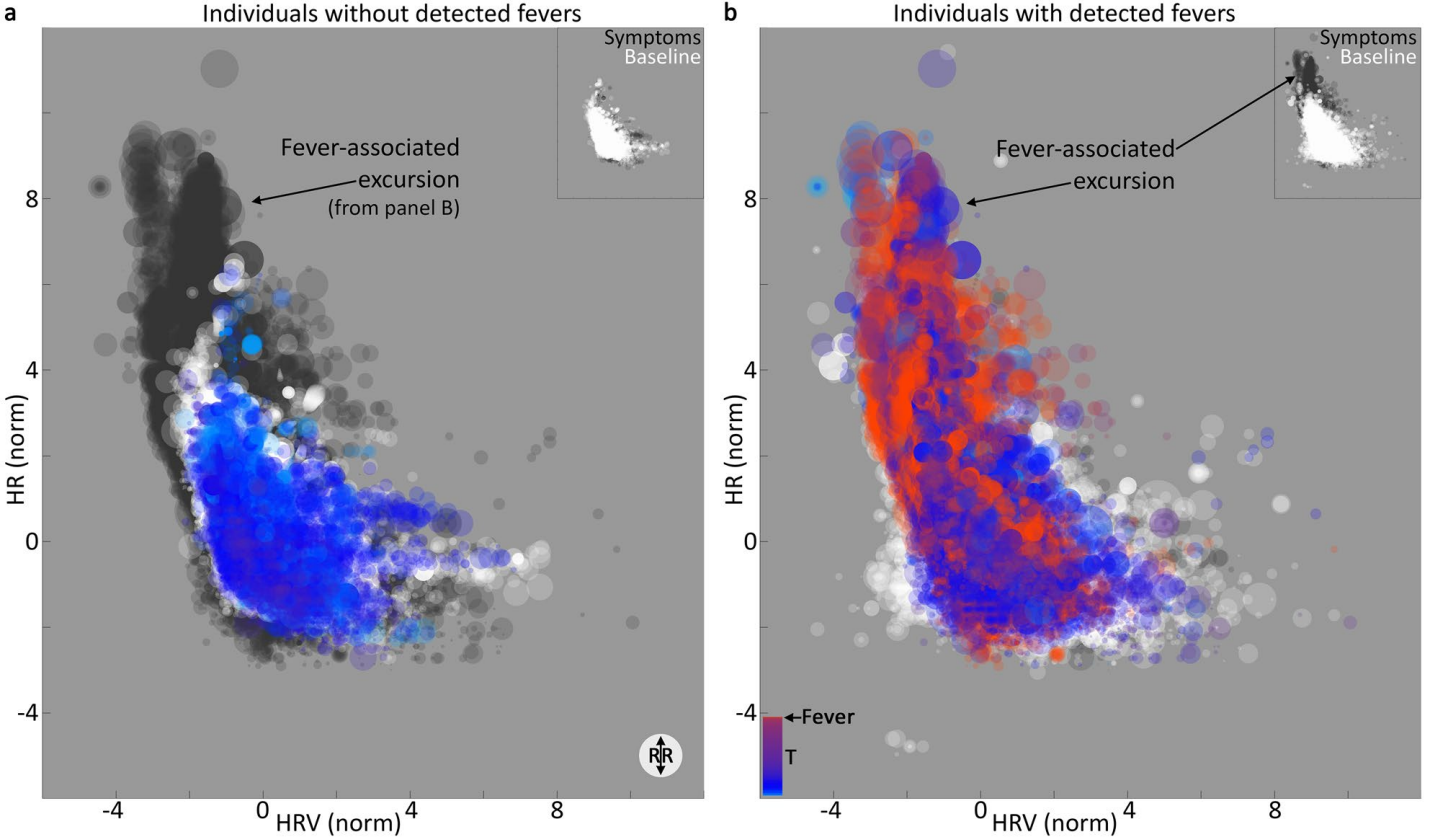
Elevated temperature is clearly detectable, but only loosely correlated to changes in HR, HRV, and RR. All time points for all individuals with original (not interpolated) measurements from temperature (T), heart rate (HR), HR variability (HRV), and respiration rate (RR) sorted into those not showing fever-like days during their reported symptom window (**a**) and those showing fever-like days during their reported symptom windows (**b**). Baseline points: white. Symptom windows: color (bar in lower left, **b**, proportional to T, max set to threshold of digital biomarker for nighttime fever detection). Dark background points: symptom window from opposite group for comparison. Dot size proportional to RR. Insets: symptom window (dark) and baseline (white) points for that panel. NB: despite being normalized by the mean of baseline day min and max values as described for temperature (see “Methods”), HR and HRV show wide ranges, reflecting large day-to-day variance within individuals. Larger and redder spots are more apparent in the “fever-associated excursion” but not restricted to this region, reflecting the lack of strong correlation across variables. Both panels use identical axes as well as scales for color and size.

Our visual examination of fever-like days also revealed substantial heterogeneity, both inside and outside the reported symptom windows. Despite a clear average of increased T min and max associated with reported fevers (Fig. 1), the exact trajectories of temperature deviation showed substantial variance across individuals and episodes (e.g. Supp 2A,B). Consistent with this observation, correlations across variables yielded low overall *r*-values (Supp 2C); the highest correlations are between cardiac variables (Supp 2C, circled), which are expected to be more related than other pairings due to the fact that increased sympathetic nervous system tone tends to both elevate HR and decrease HRV^12^. All individuals show a similar loose cluster of baseline ranges in the four variables assessed: T, HR, HRV, RR (Fig. 2, white points). Relative to individuals for whom fever-like days were not detected during their reported symptom window (Fig. 2a), individuals with detected fever-like days in their reported symptom window tended to have elevated temperature in a fever-associated excursion from this baseline cluster, in which HR increased and HRV decreased (Fig. 2b). Elevated temperature also occurred within baseline ranges of HR and HRV (Fig. 2, red veins within baseline-cluster-defined space). RR also tended to be higher in this fever-associated excursion space, though again, the linear correlation values were not high.

### Is there evidence that continuous data could feasibly be used to develop fever prediction capabilities?

**Figure 3:**
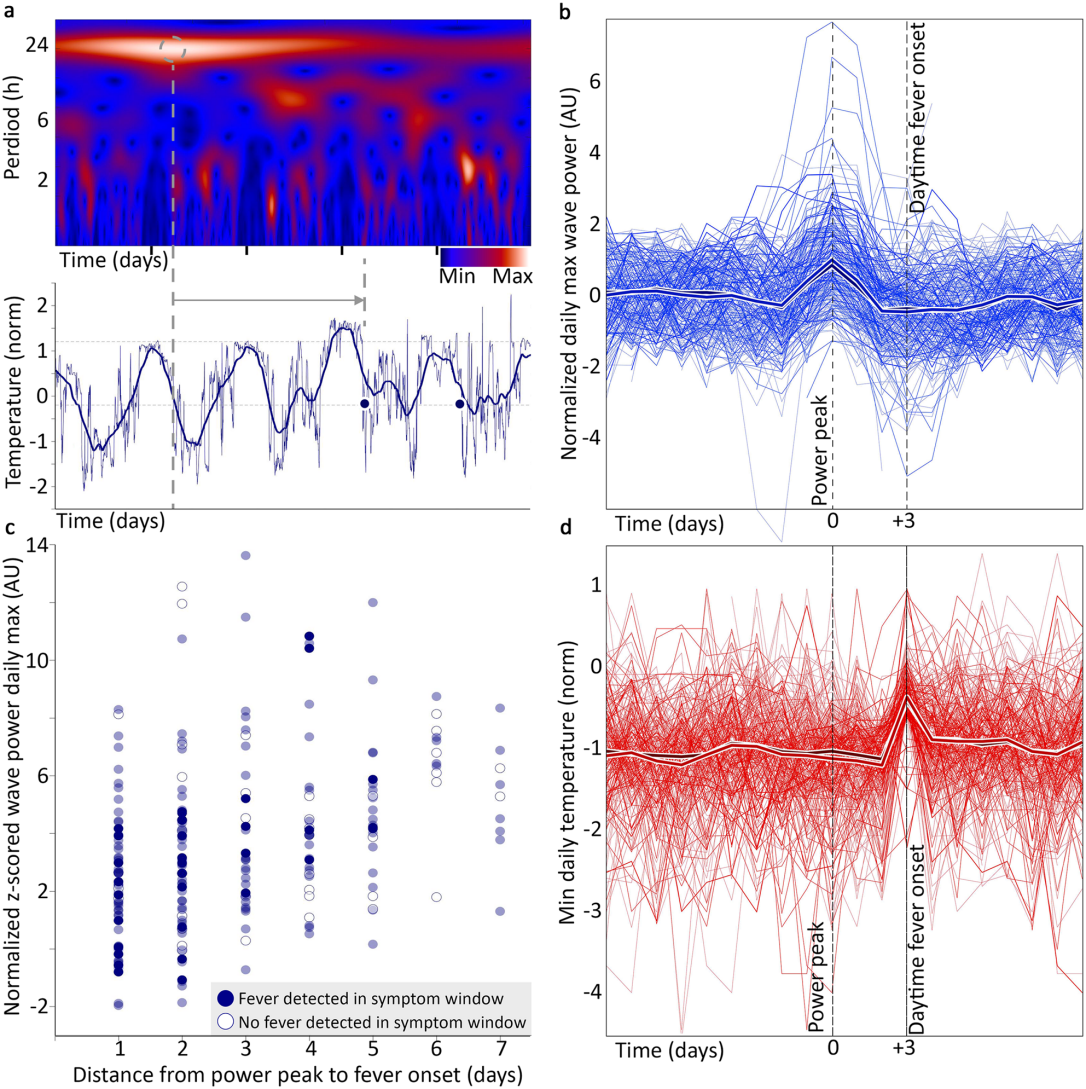
Use of signal-processing allows identification of more complex digital biomarkers in continuous data. Wavelet frequency decomposition (**a**) provides a frequency-by-time surface usable for feature extraction from continuous data. We found that 226/244 daytime fever-like events as detected by digital biomarker (**d**, aligned by onset) are preceded by a relative peak in the power of the ~ 22–26 h frequency band (**b**, all instances aligned by peak; mean: lighter fat line; median: darker fat line); example peak and alignment to fever-like day 2 days later (**a**, above to below; dots are temperature minimums for days with daytime fever threshold exceeded). The relative height of the wavelet peak correlates loosely but significantly (*r* = 0.36, *p* = 1 × 10^–7^) with time to fever onset (**c**). Hollow dots: points from individuals without detected fever-like days during reported symptom window; filled dots: points from individuals with detected fever-like days during their reported symptom window. Transparency highlights where points overlap and cluster.

Predicting onset of fever-report by identifying preceding T excursions is different than detecting the T excursions themselves. Destabilization of circadian rhythms is a risk factor for illness, and may be an early sign of physiological disruption^13–15^. The continuous nature of the temperature observations allowed us to use frequency decomposition to identify changes in approximately circadian power (ACP) that suggest the capability of these data to generate features useful for fever prediction efforts. Using a continuous wavelet decomposition (Fig. 3a), we extracted ACP bands (see “Methods”). Consistent with our hypothesis, we found unique ACP peaks (Fig. 3b) within one week before the onset of 226 of the 244 day time fever-like episodes that were detected in the data set (93%, Fig. 3d). Eighteen daytime fever-like days did not have a unique ACP peak preceding them (7%). We found a significant positive correlation between the ACP peak height and number of days until the fever-like day (*r* = 0.36, *p* = 1 × 10^–7^, Fig. 3c). The mean distance was 3 days prior to onset of daytime fever-like event, and ranged from 1–7 days, which was our assigned boundary.

## Discussion

These findings provide proof-of-concept for the feasibility of wearables-based temperature sensors to support productive research into fever-associated illness, within individuals and across distributed populations. First, our findings refute previous concerns that distal body temperature is too different from tympanic temperature to be useful in detecting fevers^7^. This previous claim was based on single time-point measures, and so the difference highlights the importance of longitudinal, high temporal-resolution data gathered within individuals. We used distal temperature data from wearables to detect changes in daily rhythms, and disturbances of thermoregulation that correspond with self-reported fevers. These data were suitable for the construction of digital biomarkers, which, when applied to the total population, had two encouraging findings. First, physiological differences from pre- to in-symptom window were stronger after re-sorting from “by reported fever” to “by digital biomarkers” (defined in “Methods”). This sign of enhanced sorting is consistent with the limited accuracy of self-reported symptoms in detecting physiological changes^16–18^. It supports the hypothesis that some fever-like events may go unreported or unnoticed without being truly asymptomatic; wearables therefore may contribute to identifying rates of asymptomatic as opposed to unreported illness, of special importance in the COVID-19 pandemic. An important limitation here is the lack of serology or other ground-truthing measures to confirm individuals’ conditions. Rather than pushing the utility of these specific digital biomarkers in detecting conditions such as COVID-19 disease, this manuscript is meant to encourage future research and applications; ideally development of such biomarkers would be coupled to symptom report and further supported by serology or other physiological confirmations of specific conditions.

Second, the majority of participants exhibited temperature anomalies prior to symptom reports (38/50), which could be flagged for investigation by comparison across other physiological variables (increased HR and RR, decreased HRV). Specifically, upon viral or bacterial infection, innate immune cells secrete proinflammatory cytokines, which alter central nervous system firing rates of warm-sensing neurons in the hypothalamus^2,19,20^. This process results in an increase in the temperature steady state, which is generally adaptive in fighting infection^2,19^. Moreover, it is accompanied by peripheral blood vessel vasoconstriction or vasodilation as a negative feedback mechanism, which conserves or releases heat (respectively), in order to help regulate temperature^19,21^. Hence, a multi-metric digital biomarker combining temperature and PPG-derived information would appear likely to provide superior illness prediction than single-signal models. To the extent these profiles systematically differ by condition, they make time series temperature, heart rate, heart rate variability, and respiratory rate data useful for early-stage indicators in illness detection and determination. Contextual information, obtained via this multi-signal profiling, may more effectively capture the dynamic physiological mechanisms that respond to infection than temperature alone. Specific studies to identify such high-dimensional biomarkers in specific pathologies (e.g. COVID-19 and other viral infections, physical exhaustion, etc.) should be supported. We believe these findings additionally show feasibility in support of investigations into the difference between “asymptomatic” and “cryptic” unnoticed, but nevertheless symptomatic, illnesses.

To date, most wearables do not use temperature sensors. This should be re-examined, as temperature contains rich physiological information^8,22–24^; distal temperature is not well mapped, but has been shown to include cues to context like daily and ultradian rhythms^23^. Moreover, the relationships across individual variables through time are not simple linear functions, and so inference of one from another (as in, projecting fevers from HR) is not a trivial problem. Additionally, the continuous nature of these data allow for modeling of these complex relationships, and are amenable to signal process approaches. These approaches allow for richer feature detection, expanding the kinds of patterns that may be visible and put to work for illness-related research and applications. It is for this last reason that, upon completion, TemPredict is hosting all data at UCSD as a curated research object to enable collaborative discovery efforts.

Despite our positive feasibility findings, we also found that major complexities lie between this work and the invention of COVID-19 detection algorithms that would be robust across individuals. The key challenges can be summarized as follows: people are different, and so are physiological systems. Taking examples displayed within this manuscript, the amplitude of daily rhythm, stability across days, correlation across variables, and stability of those correlations all change within and across individuals, both in and out of reported illness. Data from large populations willing to share information about their demographics, habits, and health will be needed to appropriately model these various flavors of variance if the COVID-19 component of those patterns is to be identifiable across such different baselines. Given the real costs associated with biases in large data endeavors^25–29^ where health is concerned, the most robust outcomes are likely to require these variance maps if they are to be useful across heterogeneous populations. Additionally, while the quality of the data is determined by the design of the hardware in large part (including ease of user compliance, comfort, etc.), here more than half our first responses could not be used due to user choices. The majority were failure to generate data (not wearing the wearable, or offering observations about illnesses before the user had a wearable) or failure to communicate critical details (wrong contact / account information so that looking up data was impossible). The resulting initial 50 cases were extremely useful, and we anticipate many more findings coming from the now ~ 1000 times larger TemPredict cohort and other ongoing large-scale studies. Nevertheless, our findings bolster the need for social research into fair wearable access and personal data trust (from the user perspective, as opposed to technical security issues), as well as efforts at data literacy education so that people (users, clinicians, policy makers, etc.) can understand the potential and the pitfalls within the generation and use of large, user-driven health data sets.

## Conclusions and implications for future work

This work shows the feasibility of gathering fever-related information from distributed populations using wearables. Our findings also suggest that the success of attempts to identify COVID-19 with specificity from wearables will require multiple physiological variables for corroboration. By coupling the wearable data to an online questionnaire, we further demonstrate the relative efficiency of gathering both physiology data and health-relevant “labels” or outcomes for research. Many caveats follow these feasibility endorsements. This work is only the proof of concept to support a great deal of future work. We see great potential for public health advancements from distributed physiological information systems, and we hope the caveats encourage deeper engagement and creative problem solving. The combination of many potential conditions across many kinds of people suggests large and sustained efforts and community engagement will be critical for this and related efforts to succeed. Best practices need to be developed to grow the potential of distributed, participatory, wearable-enabled research into a reality that is stable, safe, and productive for all parties.

## Methods

### Participants

**Figure 4:**
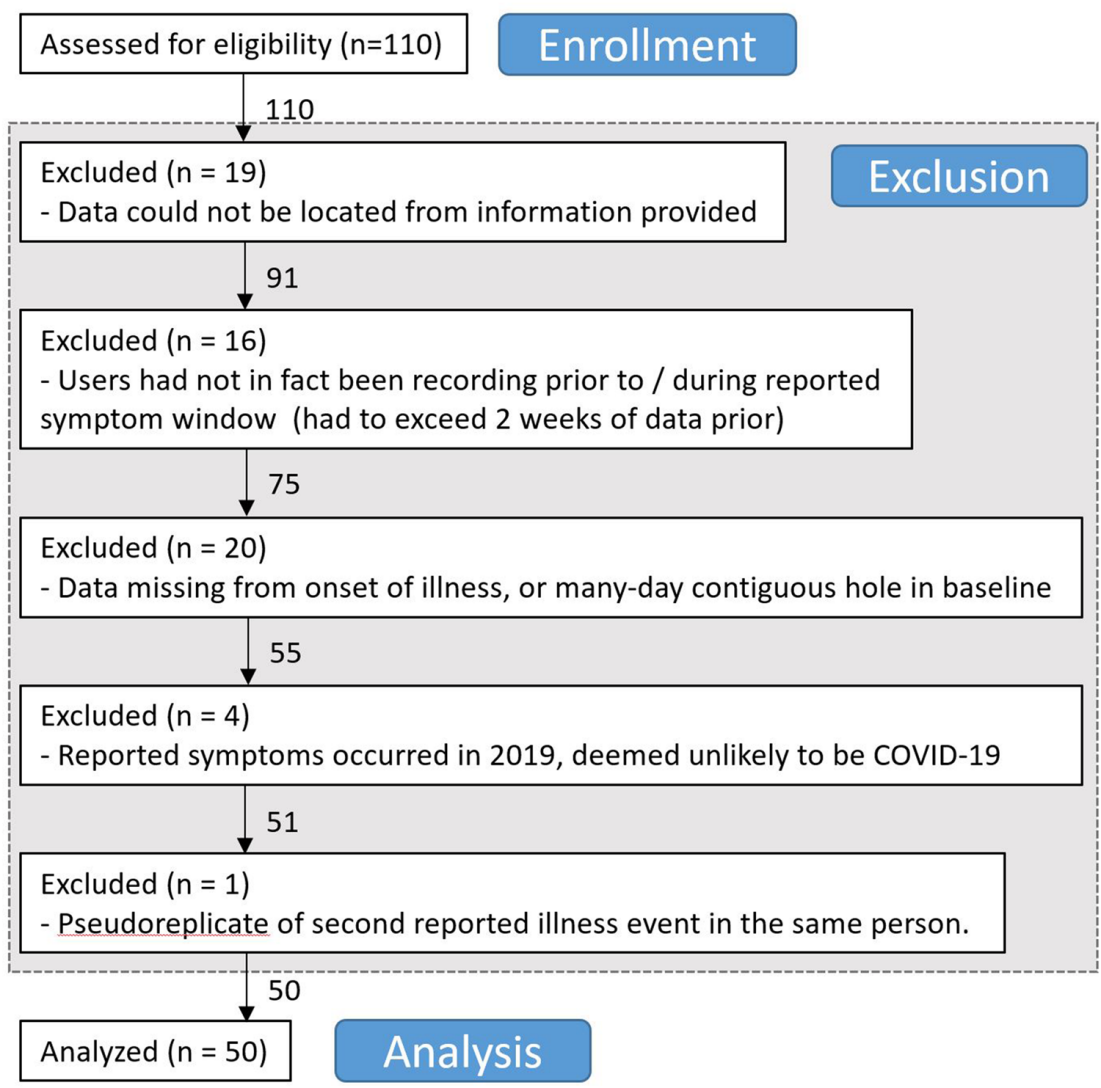
CONSORT (consolidated standards of reporting trials) format flow chart for exclusion of cases from analyses. Although neither this effort nor TemPredict are clinical trials, we show exclusion of participants from the larger dataset for ease of interpretation.

Overall Inclusion criteria for TemPredict include: (1) being 18 years of age or older, (2) possessing an Oura smartring that pairs with the Oura App on a user’s smartphone, and (3) informed consent given for study participation. Participants in these analyses possessed Oura smartrings prior to TemPredict. We did not compensate participants for their participation. All participants completed an online consent process delivered via a secure Qualtrics survey platform under a UCSF license, which allowed respondents to download PDF consent forms and indicate consent by responding to questions. Participants completed an intake survey that collected demographic information and information on any COVID-19 diagnoses received prior to enrollment. This survey included instructions for how participants could provide TemPredict researchers with access to their Oura ring wearable data. Once enrolled, participants accessed a daily symptom survey through the Oura App on their smartphone. This daily survey collected information on COVID-19 symptoms and (if applicable) if and when the participants received any COVID-19 diagnoses or completed any relevant testing. Participants for this analysis were selected from 110 initial individuals who enrolled in TemPredict and who reported that they had experienced COVID-19 symptoms prior to enrolling in the study (Fig. 4); these participants then filled out a one-time questionnaire about their COVID-19 symptoms (see “Self-report measures” below). The University of California San Francisco (UCSF) Institutional Review Board (IRB) approved all study procedures, and all work was carried out in accordance with these approvals, and all relevant regulations.

### Physiological measures

We collected all physiological measures using the Oura ring, a commercially available wearable sensor device (Oura Health, Oulu, Finland). The Oura ring connects to the Oura App (available from the Google Play Store or the Apple App Store) via Bluetooth. Users wear the ring on any finger and can wear the ring continuously in wet and dry environments while doing activity that a human hand can tolerate. The Oura ring assesses temperature using a negative temperature coefficient (NTC) thermistor (non-calibrated, resolution of 0.07 °C) on the internal surface of the ring. The sensor is programmed to register skin temperature readings from the palm side of the finger base every minute. The Oura ring assesses heart rate, heart rate variability, and respiration rate by extracting features from a photoplethysmogram (PPG) signal generated at 250 Hz. Specifically, respiration rate (RR) observations are stored at 30 s resolution; heart rate (HR) is provided as the mean derived per 5 min of inter-beat interval (IBI); heart rate variability (HRV) is provided in the form of RMSSD derived per 5 min of IBI; all of these metrics are generated on device, and the raw PPG is not continuously recorded or stored for analysis.

### Self-report measures

At intake, participants report on demographic factors including age, race/ethnicity, educational background, and country/state of residence. Participants in these analyses reported on the date that they first felt ill (“*what date did you first notice symptoms?*”), the date they believe they had recovered (“*what date would you say you recovered (defined as major symptoms resolved and feeling almost like your usual self)?*”), and symptoms they experienced (“*When you suspected (or confirmed) that you had COVID-19, which of these symptoms did you experience?*”). For these analyses, we derived “symptom window” using these dates, and the only symptom isolated in these analyses was “fever”.

### Data preparation

We prepared and analyzed data using Matlab 2019b. We generated visualizations in Matlab and formatted and arranged them in Adobe Photoshop. Of the 110 first participants to respond to our request for information about pre-TemPredict episodes of possible COVID-19 cases, 20 had such large windows of missing Oura data that we deemed impossible any comparison of baseline data to sickness onset; 19 could not be located in the Oura database (presumably due to typos in the information they reported to us); 16 did not register Oura data prior to / during their reported episode, or began so close before that they could not generate a prior baseline (threshold set to 14 days prior to first day of symptom report); 4 occurred before 2020, and so were deemed unlikely to be COVID-19; and 1 was a second symptom episode from the same user, and we opted to keep out pseudoreplication from our comparisons. These exclusions brought us to our first 50 usable episodes.

We aligned data from episodes of reported COVID-19 cases by time to the day of symptom report onset. Data include heart rate (HR), heart rate variability (HRV), temperature (T), and respiratory rate (RR). We limited all variables to a window starting 45 days prior to the first day of symptom report, and then 20 days subsequent, counting inclusively of the first day with reported symptoms (65 days total, with all subjects aligned by the day of symptom report onset). Across this dataset, all data were interpolated to 1 min resolution using a linear interpolation. We indexed “baseline” as all data in days 1–40 pre-symptom report onset. We indexed “symptom window” as each individual’s window of reported symptoms. For plots representing populations, the mean symptom window duration is used in plotting (mean 9.3 days), but the analyses are carried out within-individual using that individual’s symptom window.

To examine the overlap of ranges across individuals, we calculated T mean and standard deviation for each individual over either their baseline window or their symptom window + 1 day prior to symptom onset. To understand how changes in temperature correlated to symptom window, data were transformed into z-scores, then representative daily minimum and maximum were generated by taking the median of the lowest and highest 360 min per 24 h window across the 65 days. In this way, minimum and maximum values were less dependent on outlier events or acute extremes. The whole of the dataset was then normalized so that the mean of the baseline daily min and max for all four physiological variables for each individual ranged from − 1 to 1. Fever threshold was determined to be daily max > 1.2 or daily min > − 0.2. *NB*: no attempt was made to optimize these thresholds beyond 1 significant digit, as the goal was to test feasibility of using such data to generate digital biomarkers, and to avoid over-stating claims about precision from this initial cohort, which might encourage a reader to see these values as solutions, rather than proofs of concept.

After dividing users into two groups based on the presence or absence of self-reported fever (n = 38, n = 12, respectively), we used our digital biomarkers (temperature min and max thresholds) to re-sort subjects by presence or absence of detected fever-like day within their reported symptom window (n = 42, n = 8, respectively). We then calculated group mean change between negative and positive fever groups in both sortings as difference between baseline mean and the mean of the first week of symptom report for all variables. Difference between groups was calculated using a nonparametric Kruskal Wallace test, with Tukey–Kramer post hoc comparison.

Clustering across variables was carried out only on those moments during which original observations for all variables were present—this was limited to 5 min intervals (the storage rate for the mean HR and RMSSD), mostly at night, due to the sleep-focused PPG activation used by the device manufacturer. Pearson’s correlations were run on the simplified dataset of daily min and max values across all variables and all days.

We conducted wavelet analysis as previously reported^22,30^. Briefly, in-house code for wavelet decomposition modified from the “Jlab” toolbox and from code developed by Dr. Tanya Leise^31^, using the morse wavelet (b = 5, g = 3)^32^. Because wavelet transformations (WTs) exhibit artifacts at the edges of the data being transformed, we excluded events identified in the WTs if they were within 2 periods from the edge of the data (e.g., 48 h at the 24 h period). To quantify 24 h power, the maximum for each timepoint (1-min resolution) was identified in the band between 22–26 h periodicity, and the mean taken across 1440 min (24 h) windows. For comparison to the onset of day-time fever-like events detected by our digital biomarker, peaks in wavelet power were defined as the highest of surrounding daily maximums. Note that data pairs (peak:fever-like day) were omitted when peak resulted from missing data or artifacts within data. To ensure data quality, all cases were reviewed by eye and peaks resulting from missing or artifactual data were removed. Data reported are subsequent to this cleaning, and so do not reflect artifactual peaks.

## Supplementary information

Supplementary Information

## Data Availability

Data used in this manuscript, and associated Matlab code, are available at the UCSD Research Data Library under the following DOI information: Smarr, Benjamin L.; Aschbacher, Kirstin; Fisher, Sarah; Chowdhary, Anoushka; Dilchert, Stephan; Puldon, Karena; Rao, Adam; Hecht, Frederick M.; Mason, Ashley E. yy. Data from: Feasibility of continuous fever monitoring using wearable devices. UC San Diego Library Digital Collections. https://doi.org/10.6075/J0ZW1JFX.

## References

[1] Definitions of Symptoms for Reportable Illnesses | Quarantine | CDC (2019) (June 15, 2020).

[2] Walter EJ, Hanna-Jumma S, Carraretto M, Forni L (2016). The pathophysiological basis and consequences of fever. Crit. Care.

[3] Zhou F (2020). Clinical course and risk factors for mortality of adult inpatients with COVID-19 in Wuhan, China: a retrospective cohort study. Lancet.

[4] Mouchtouri VA (2019). Exit and entry screening practices for infectious diseases among travellers at points of entry: looking for evidence on public health impact. Int. J. Environ. Res. Public. Health.

[5] J. Bogaisky, Tech that scans people for fever in big demand amid coronavirus crisis. Boosting Wuhan Company. *Forbes* (June 15, 2020).

[6] K. Gostic, A. C. Gomez, R. O. Mummah, A. J. Kucharski, J. O. Lloyd-Smith, Estimated effectiveness of symptom and risk screening to prevent the spread of COVID-19. *eLife* **9**, e55570. 10.7554/eLife.55570 PMC7060038 32091395

[7] Chen H-Y, Chen A, Chen C (2020). Investigation of the impact of infrared sensors on core body temperature monitoring by comparing measurement sites. Sensors.

[8] Grant AD, Wilsterman K, Smarr BL, Kriegsfeld LJ (2018). Evidence for a coupled oscillator model of endocrine ultradian rhythms. J. Biol. Rhythms.

[9] Maijala A, Kinnunen H, Koskimäki H, Jämsä T, Kangas M (2019). Nocturnal finger skin temperature in menstrual cycle tracking: ambulatory pilot study using a wearable Oura ring. BMC Womens Health.

[10] Baker FC, Driver HS (2007). Circadian rhythms, sleep, and the menstrual cycle. Sleep Med..

[11] Abbasi J (2017). Wearable Digital Thermometer Improves Fever Detection. JAMA.

[12] U. Rajendra Acharya, K. Paul Joseph, N. Kannathal, C. M. Lim, J. S. Suri, Heart rate variability: a review. *Med. Biol. Eng. Comput.* **44**, 1031–1051 (2006). 10.1007/s11517-006-0119-0 17111118

[13] Foster RG (2020). Sleep, circadian rhythms and health. Interface Focus.

[14] Boyko Y, Jennum P, Toft P (2017). Sleep quality and circadian rhythm disruption in the intensive care unit: a review. Nat. Sci. Sleep.

[15] Murakami M, Tognini P (2019). The circadian clock as an essential molecular link between host physiology and microorganisms. Front. Cell. Infect. Microbiol..

[16] Bradford VP, Graham BP, Reinert KG (1993). Accuracy of self-reported health histories: a study. Mil. Med..

[17] Kim Y-Y (2017). Level of agreement and factors associated with discrepancies between nationwide medical history questionnaires and hospital claims data. J. Prev. Med. Pub. Health.

[18] Barbara AM, Loeb M, Dolovich L, Brazil K, Russell M (2012). Agreement between self-report and medical records on signs and symptoms of respiratory illness. Prim. Care Respir J. J. Gen. Pract. Airw. Group.

[19] Garami A, Steiner AA, Romanovsky AA (2018). Fever and hypothermia in systemic inflammation. Handb. Clin. Neurol..

[20] A. Blomqvist, D. Engblom, Neural Mechanisms of Inflammation-Induced Fever. *Neurosci. Rev. J. Bringing Neurobiol. Neurol. Psychiatry* **24**, 381–399 (2018). 10.1177/1073858418760481 PMC6047205 29557255

[21] Romanovsky AA (2004). Do fever and anapyrexia exist? Analysis of set point-based definitions. Am. J. Physiol. Regul. Integr. Comp. Physiol..

[22] Smarr BL, Zucker I, Kriegsfeld LJ (2016). Detection of successful and unsuccessful pregnancies in mice within hours of pairing through frequency analysis of high temporal resolution core body temperature data. PLoS ONE.

[23] Smarr B, Burnett D, Mesri S, Pister K, Kriegsfeld L (2015). A wearable sensor system with circadian rhythm stability estimation for prototyping biomedical studies. IEEE Trans. Affect. Comput..

[24] Smarr BL, Grant AD, Zucker I, Prendergast BJ, Kriegsfeld LJ (2017). Sex differences in variability across timescales in BALB/c mice. Biol. Sex Differ..

[25] Gianfrancesco MA, Tamang S, Yazdany J, Schmajuk G (2018). Potential biases in machine learning algorithms using electronic health record data. JAMA Intern. Med..

[26] I. Johnson, C. McMahon, J. Schöning, B. J. Hecht, The Effect of Population and “Structural” Biases on Social Media-based Algorithms: A Case Study in Geolocation Inference Across the Urban-Rural Spectrum. *CHI 17 Proc. 2017 CHI Conf. Hum. Factors Comput. Syst.*, 1154–1166 (2017).

[27] B. Glymour, J. Herington, Measuring the Biases that Matter: The Ethical and Casual Foundations for Measures of Fairness in Algorithms in *Proceedings of the Conference on Fairness, Accountability, and Transparency*, FAT* ’19., (Association for Computing Machinery, 2019), pp. 269–278.

[28] Kleinberg J, Ludwig J, Mullainathan S, Rambachan A (2018). Algorithmic fairness. AEA Pap. Proc..

[29] Obermeyer Z, Powers B, Vogeli C, Mullainathan S (2019). Dissecting racial bias in an algorithm used to manage the health of populations. Science.

[30] Leise TL (2013). Wavelet analysis of circadian and ultradian behavioral rhythms. J. Circ. Rhythms.

[31] Leise TL (2015). Wavelet-based analysis of circadian behavioral rhythms. Methods Enzymol..

[32] Lilly JM, Olhede SC (2012). Generalized morse wavelets as a superfamily of analytic wavelets. IEEE Trans. Signal Process..

