# Author Correction: Feasibility of continuous fever monitoring using wearable devices

**DOI:** 10.1038/s41598-022-08621-2

**Published:** 2022-03-15

**Authors:** Benjamin L. Smarr, Kirstin Aschbacher, Sarah M. Fisher, Anoushka Chowdhary, Stephan Dilchert, Karena Puldon, Adam Rao, Frederick M. Hecht, Ashley E. Mason

**Affiliations:** 1 Department of Bioengineering and Halicioglu Data Science Institute, University of California, San Diego, 9500 Gilman Drive MC 0412, La Jolla, CA 92093-0412 USA; 2 Division of Cardiology, School of Medicine, University of California, San Francisco, San Francisco, USA; 3 Health Data Architect, Science Team, Oura, San Francisco, USA; 4 Osher Center for Integrative Medicine, University of California San Francisco, San Francisco, USA; 5 Department of Management, Baruch College, CUNY, New York and preValio LLC, Minneapolis, USA; 6 School of Medicine, University of California, San Francisco, San Francisco, USA; 7 Department of Psychiatry, University of California, San Francisco, San Francisco, USA

Correction to: *Scientific Reports* 10.1038/s41598-020-78355-6, published online 14 December 2020

The original version of this Article contained an error in the Funding section.

“In addition, the researchers were otherwise from Oura Health Oy and that all authors, external and internal, had full access to all of the data (including statistical reports and tables) in the study and can take responsibility for the integrity of the data and the accuracy of the data analysis.”

now reads:

“BS and AEM had full access to all of the data (including statistical reports and tables) and can take responsibility for the integrity of the data and the accuracy of the data analysis.”

The original Article has been corrected.

