# Supplementary material for: Feasibility of continuous fever monitoring using wearable devices
Source: Sci Rep. 2020 Dec 14;10:21640. doi: 10.1038/s41598-020-78355-6 (PMC7736301; doi:10.1038/s41598-020-78355-6)
Supplement: Supplementary file 1 — Supplementary Information [file 41598_2020_78355_MOESM1_ESM.docx]

**Title.** Feasibility of continuous fever monitoring using wearable devices.

**Author order.** Benjamin L. Smarr^1^, Kirstin Aschbacher^2,3^, Sarah M. Fisher^4^, Anoushka Chowdhary^5^, Stephan Dilchert^6^, Karena Puldon^7^, Adam Rao^8^, Frederick M. Hecht*^9^, Ashley E. Mason*^10,11^


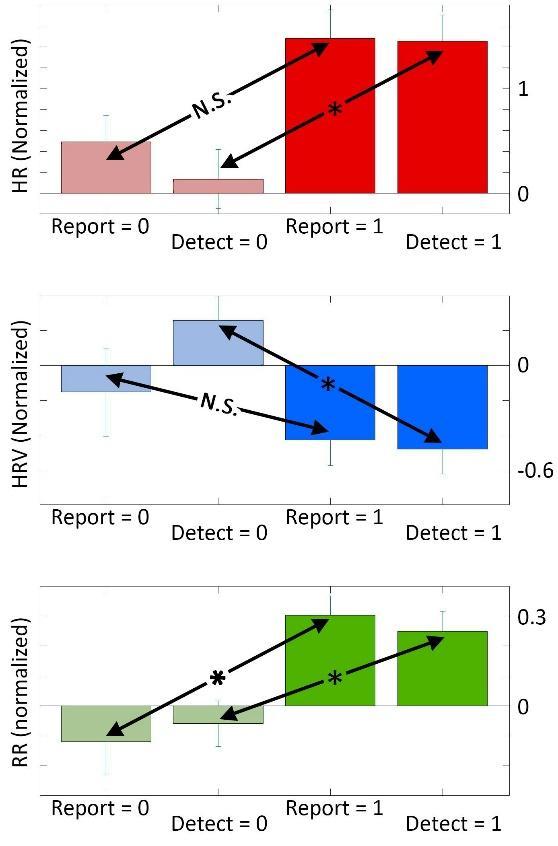
**Supplemental Information.**

**Supp 1. Use of a digital biomarker improves on symptom-report for group separation.** Sorting cases using symptom report (“Report”; 0: no fever; 1: fever) leads to a non-significant difference between the baseline (pale bars) and the first week of reported symptoms (full-color bars; error bars S.E.) for HR and HRV (N.S.) (red: HR; blue: HRV; green: RR). A significant difference in RR is present (*). Resorting individuals by digital biomarker (“Detect”0: no fever; 1: fever) results in significant differences between baseline and the first week of reported symptoms in HR and HRV (Tukey-Kramer post-hoc analysis following Kruskal-Wallis).


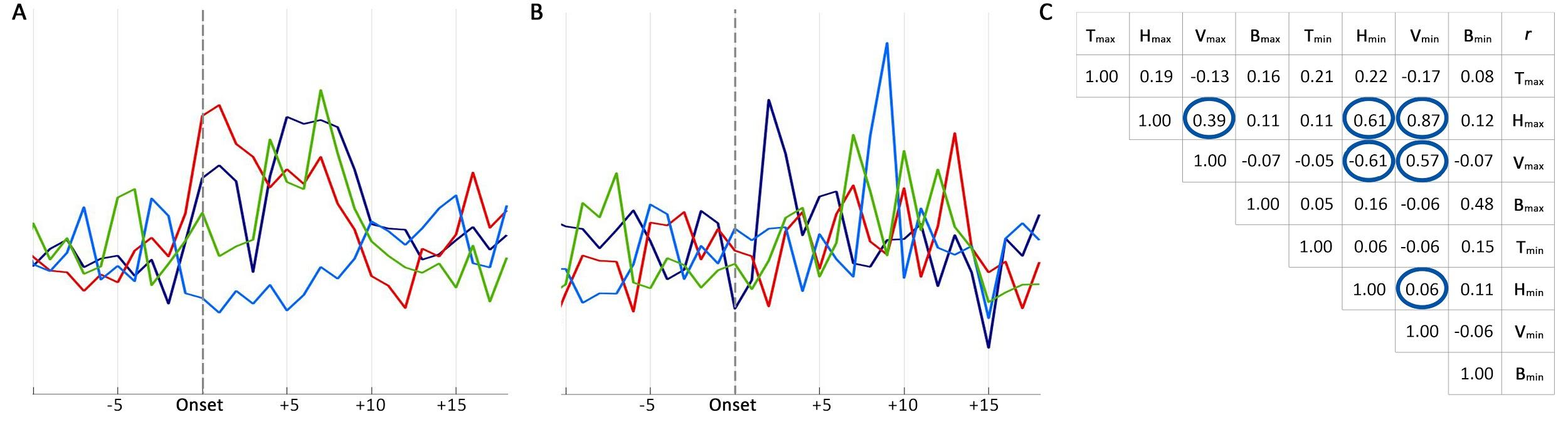


**Supp 2. Example sickness profiles show lack of strong correlation across physiological variables.** Two examples (A, B) show that although the general profile of fever-associated illness can be seen in other variables (daily maximums: Temp: dark blue; HR: red; HRV: light blue; RR: green; arbitrary units), they do not operate in unison. A: fever onset precedes symptom report, with a rise in Temp and HR associated with a decrease in HRV. NB: in the first peak HR shows the highest rise, RR the least, whereas in the second peak RR is the highest, and HR the least. B: temperature spike associated with symptom onset precedes changes in HR, HRV, and RR by 1-2 weeks. C: Pearson’s correlations of daily maximum and minimums for all variables in individuals with detected fever-like days within their symptom windows (those displayed in Fig 1B), inter-individual mean. NB: the largest absolute correlation, r = -0.72, is still not very strong; all the highest correlation values (circles) are within cardiac variables, while none correlate strongly with temp or RR, the first of which defines fever, and the combination of which are the two noted changes documented for COVID-19.
